# Supplementary figures and images for: PRMT1-mediated metabolic reprogramming promotes leukemogenesis
Source: eLife. 2025 Aug 13;14:RP105318. doi: 10.7554/eLife.105318 (PMC12349897; doi:10.7554/eLife.105318)

None

WT

WT+v1

WT+v2

Mut

Mut+v1

Mut+v2

RBM15

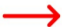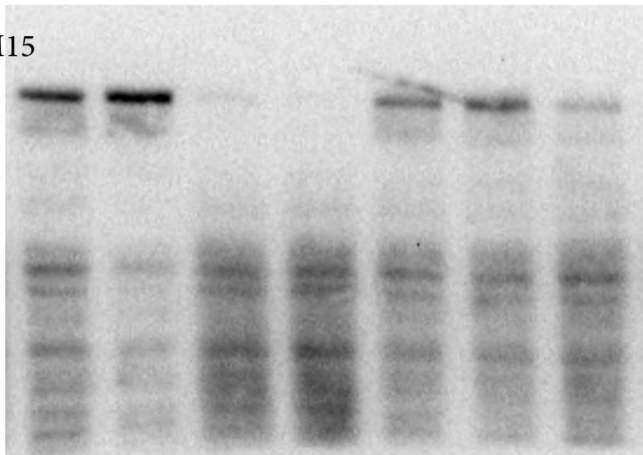

Supplement: Figure 2—figure supplement 1—source data 1. [file elife-105318-fig2-figsupp1-data1.zip › Figure 2-supplement figure 2a- labeled source data/Figure 2-supplement figure 2A-source data 8.pdf]

None WT WT+v1 WT+v2 Mut Mut+v1 Mut+v2

GAPDH

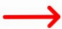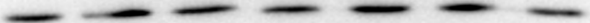

Supplement: Figure 2—figure supplement 1—source data 1. [file elife-105318-fig2-figsupp1-data1.zip › Figure 2-supplement figure 2a- labeled source data/Figure 2-supplement figure 2a-source data 5.pdf]

None WT WT+v1 WT+v2 Mut Mut+v1 Mut+v2

PRMT1

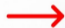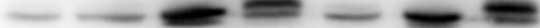

Supplement: Figure 2—figure supplement 1—source data 1. [file elife-105318-fig2-figsupp1-data1.zip › Figure 2-supplement figure 2a- labeled source data/Figure 2-supplement figure 2A-source data 7.pdf]

→  
HA

None

WT

WT+v1

WT+v2

Mut

Mut+v1

Mut+v2

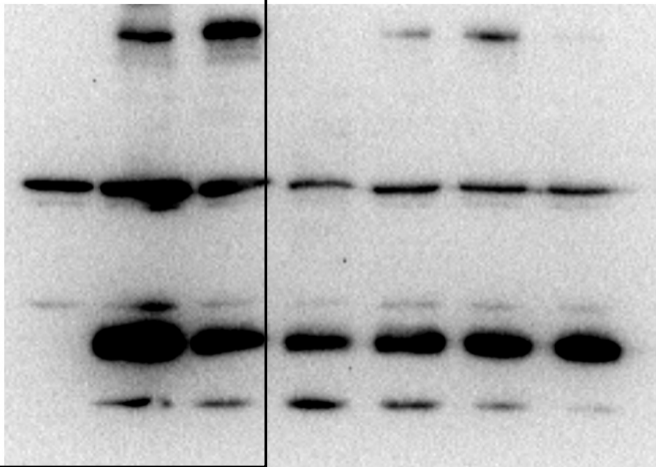

Supplement: Figure 2—figure supplement 1—source data 1. [file elife-105318-fig2-figsupp1-data1.zip › Figure 2-supplement figure 2a- labeled source data/Figure 2- supplement figure2 A- source data 6.pdf]

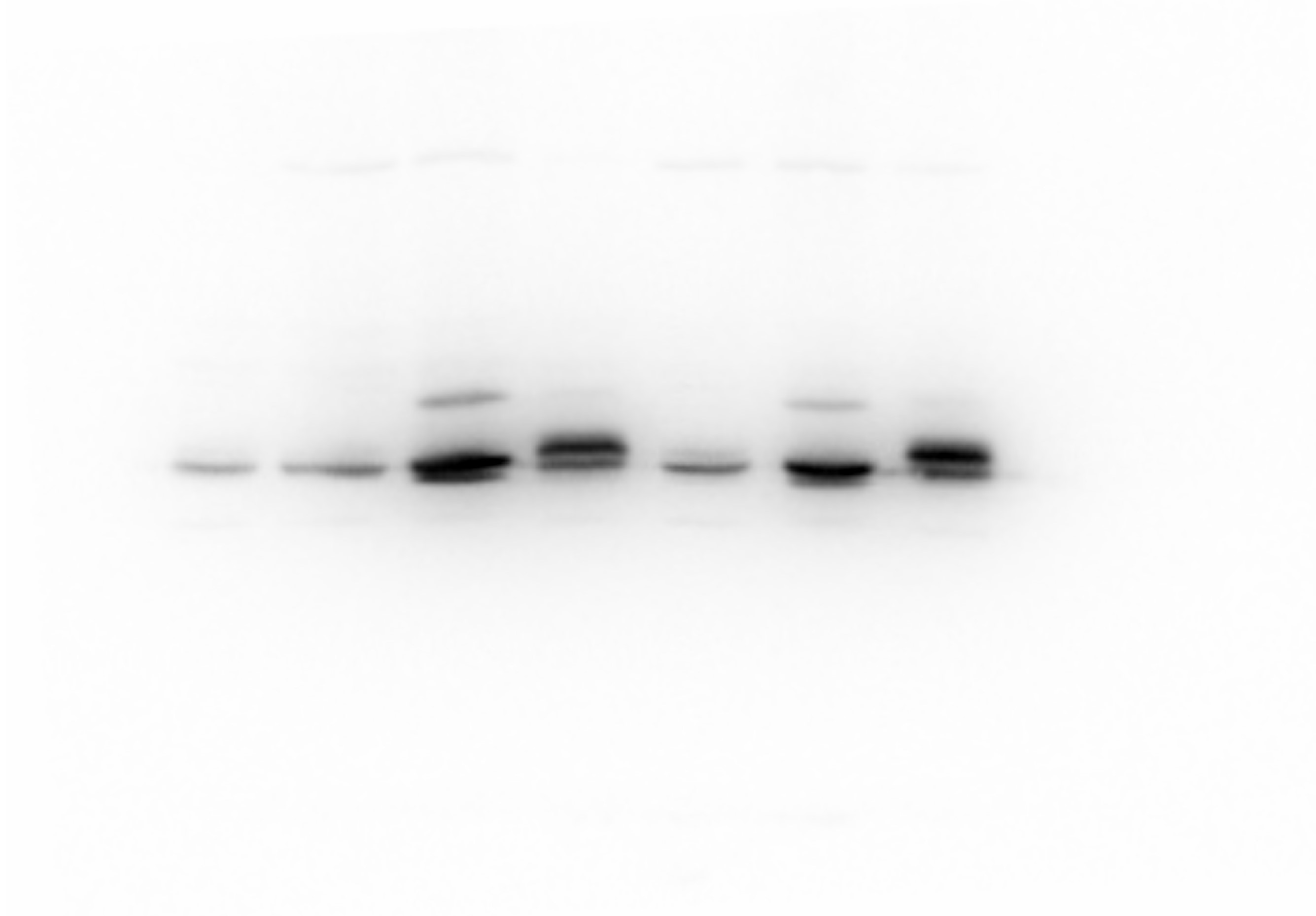

Supplement: Figure 2—figure supplement 1—source data 2. [file elife-105318-fig2-figsupp1-data2.zip › Figure 2 raw data/Figure 2-source data 3.jpg]

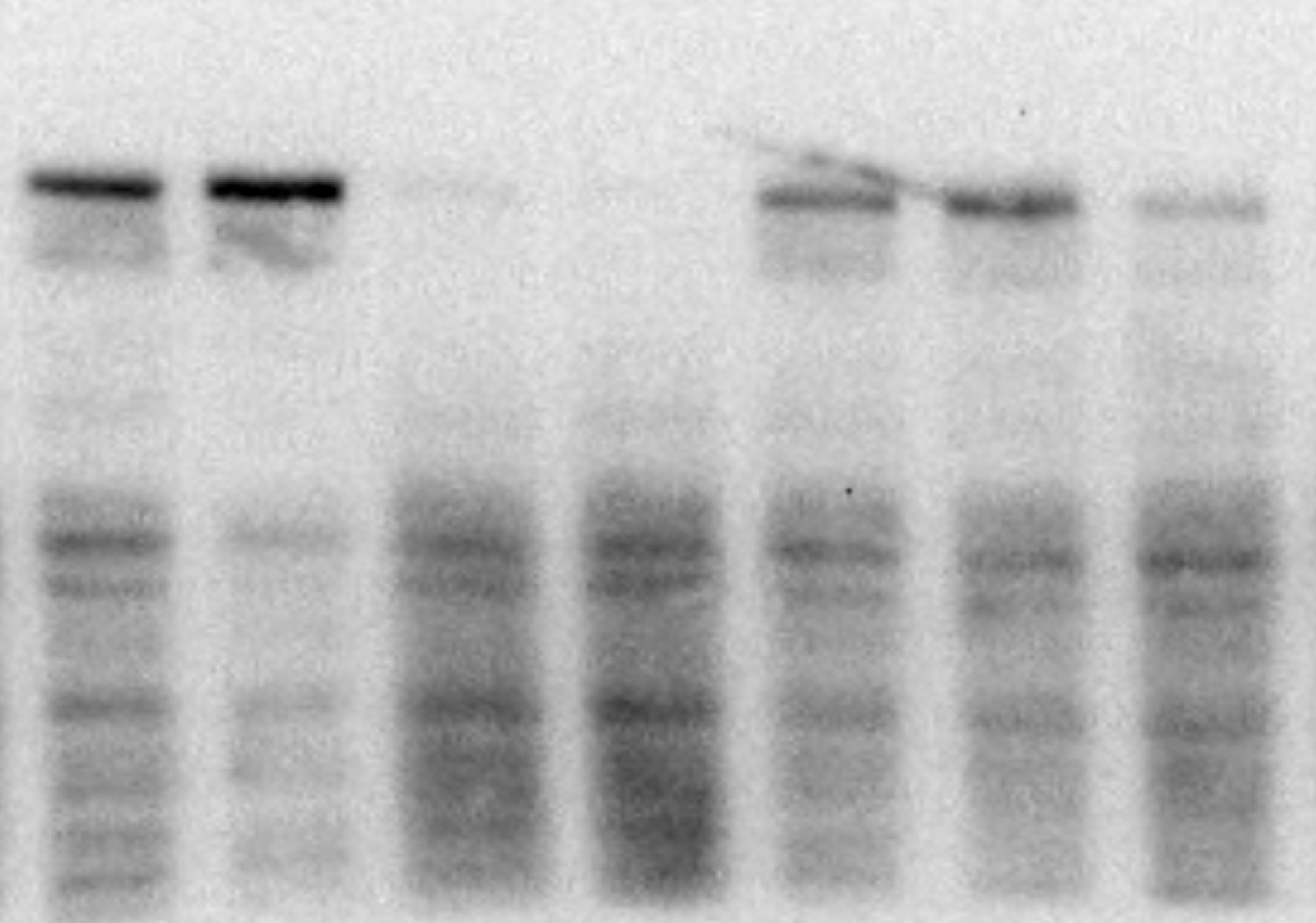

Supplement: Figure 2—figure supplement 1—source data 2. [file elife-105318-fig2-figsupp1-data2.zip › Figure 2 raw data/Figure 3-source data 4.jpg]

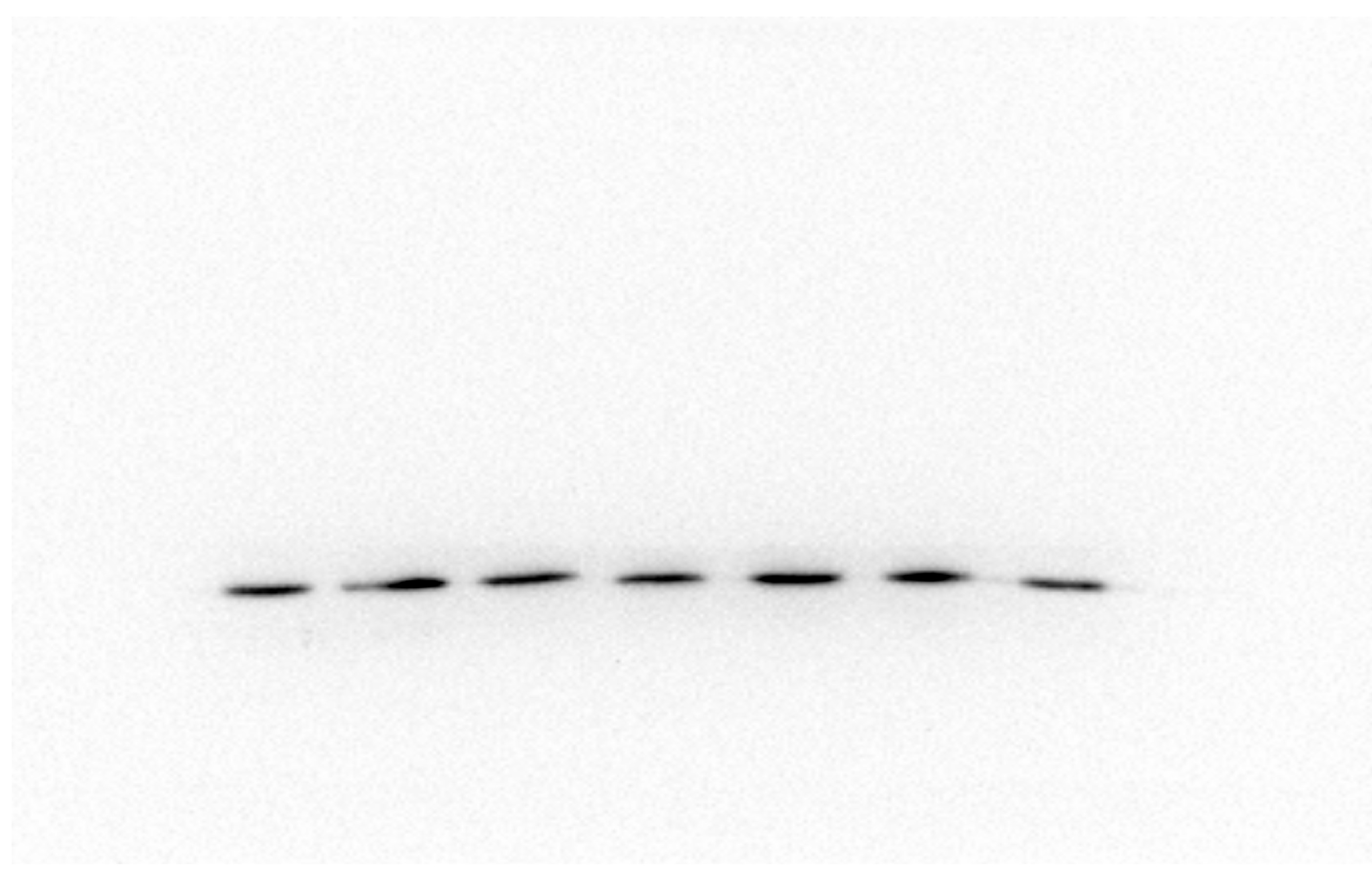

Supplement: Figure 2—figure supplement 1—source data 2. [file elife-105318-fig2-figsupp1-data2.zip › Figure 2 raw data/Figure 2-source data 1.jpg]

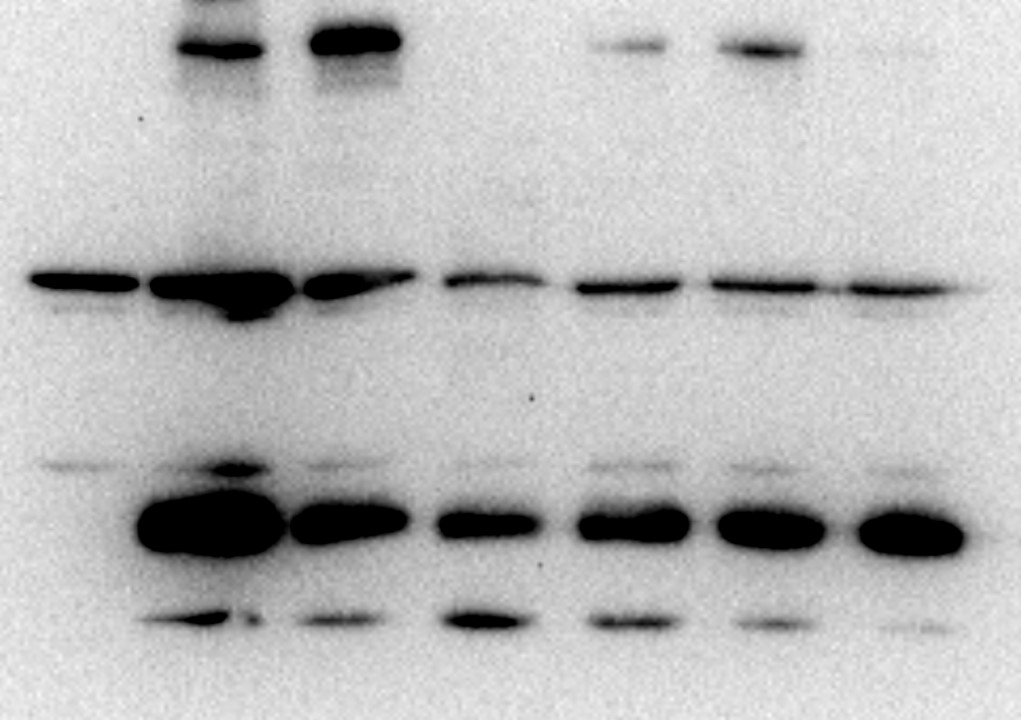

Supplement: Figure 2—figure supplement 1—source data 2. [file elife-105318-fig2-figsupp1-data2.zip › Figure 2 raw data/Figure 2-source data2.jpg]

None

WT

WT+MS

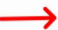

RBM15

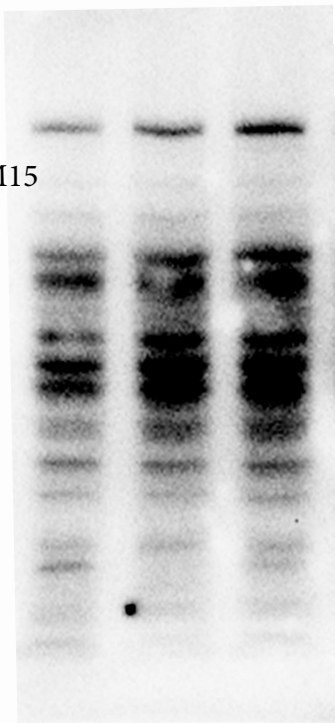

Supplement: Figure 2—figure supplement 1—source data 3. [file elife-105318-fig2-figsupp1-data3.zip › Figure 2-supplement figure 2B labeled source data/Figure 2-supplement figure 2 b-source data 8.pdf]

None

WT

WT+MS

GPADH

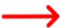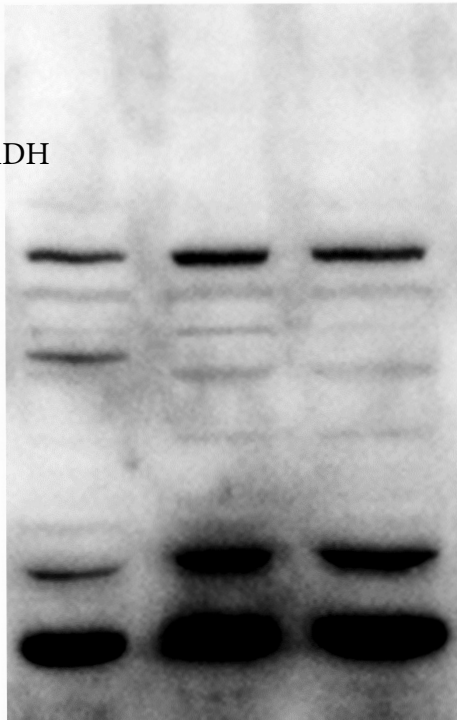

Supplement: Figure 2—figure supplement 1—source data 3. [file elife-105318-fig2-figsupp1-data3.zip › Figure 2-supplement figure 2B labeled source data/Figure 2-supplement figure 2b-source data 5.pdf]

prmt1

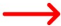

None

WT

WT+MS

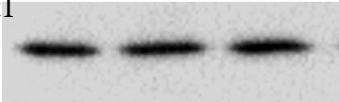

Supplement: Figure 2—figure supplement 1—source data 3. [file elife-105318-fig2-figsupp1-data3.zip › Figure 2-supplement figure 2B labeled source data/Figure 2-supplement figure 2b-source data 7.pdf]

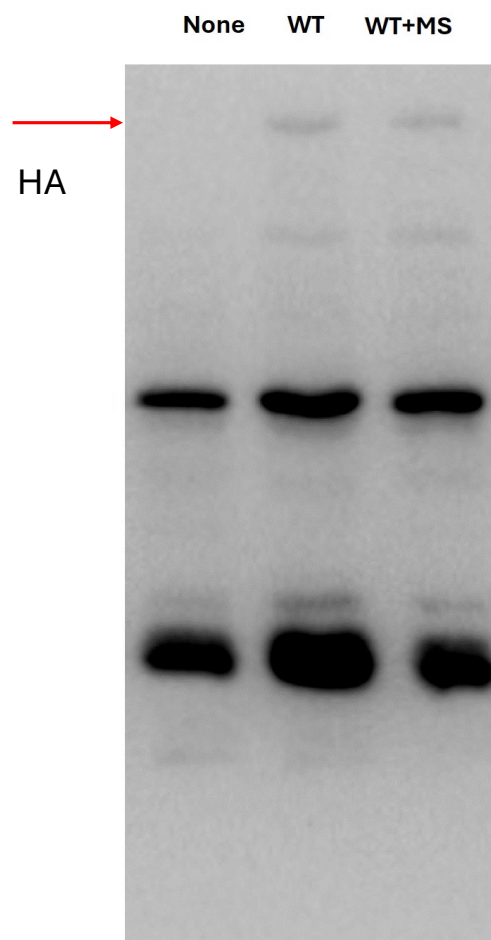

Supplement: Figure 2—figure supplement 1—source data 3. [file elife-105318-fig2-figsupp1-data3.zip › Figure 2-supplement figure 2B labeled source data/Figure 2-supplement figure 2B-source data 6.pdf]

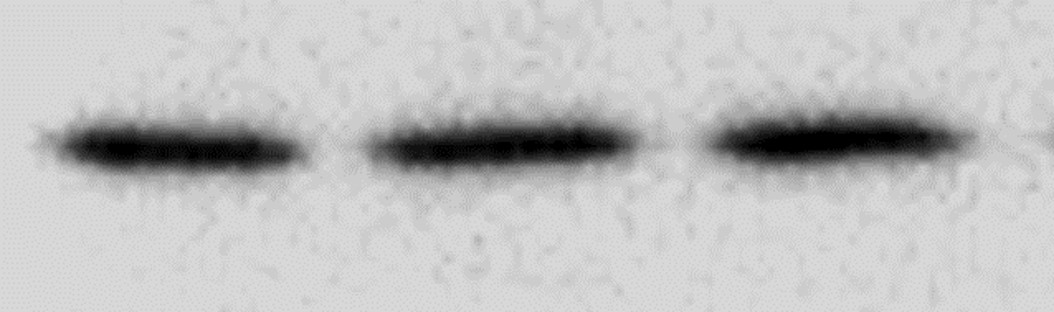

Supplement: Figure 2—figure supplement 1—source data 4. [file elife-105318-fig2-figsupp1-data4.zip › Figure 2-supplement figure 2B-source data/Figure 2-supplement figure 2 B-source data 3.jpg]

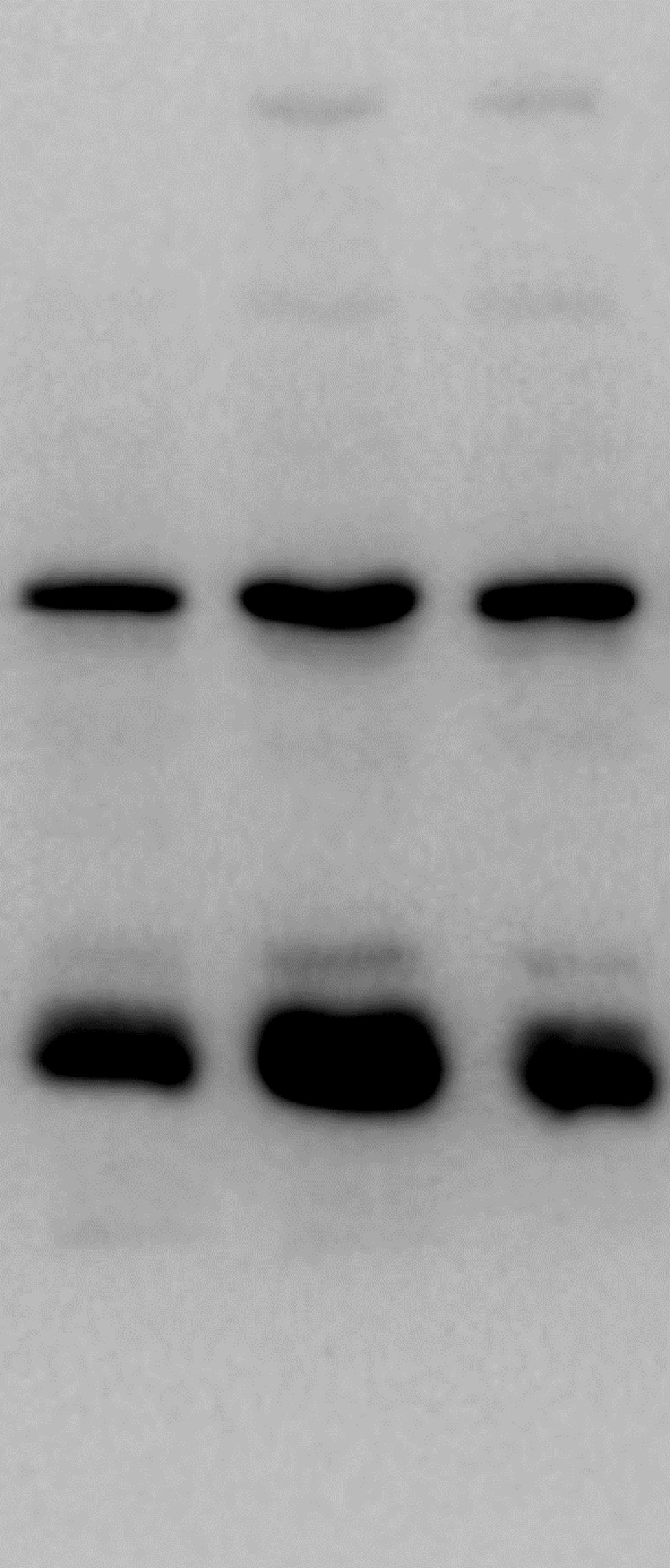

Supplement: Figure 2—figure supplement 1—source data 4. [file elife-105318-fig2-figsupp1-data4.zip › Figure 2-supplement figure 2B-source data/Figure 2-supplement figure 2 B-source data 2.jpg]

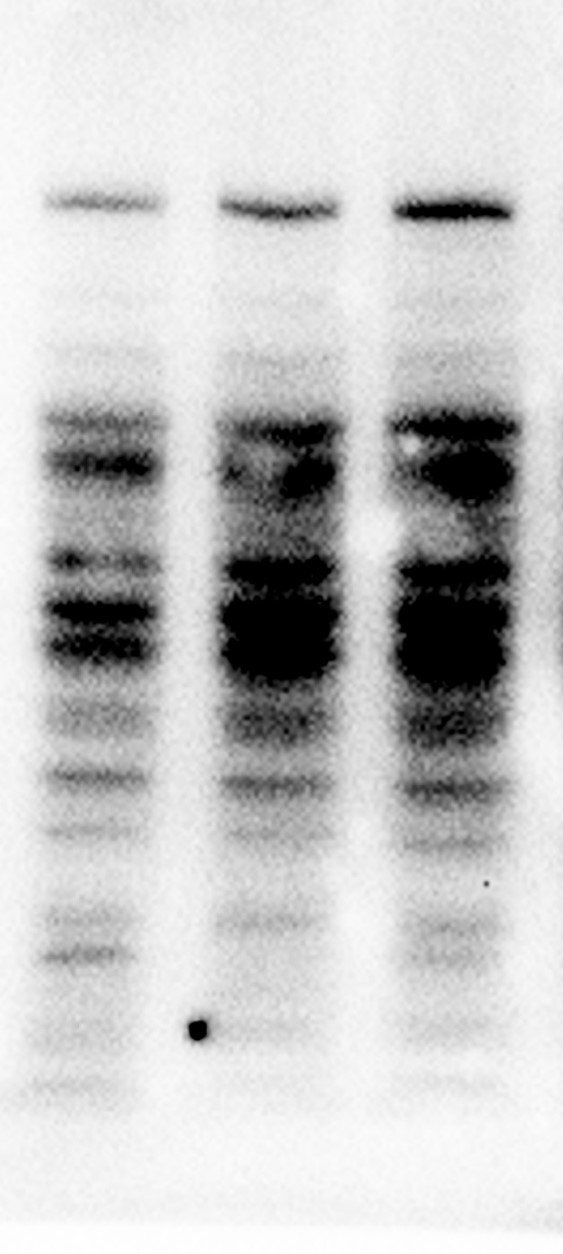

Supplement: Figure 2—figure supplement 1—source data 4. [file elife-105318-fig2-figsupp1-data4.zip › Figure 2-supplement figure 2B-source data/Figure 2-supplement figure 2 B-source data 4.jpg]

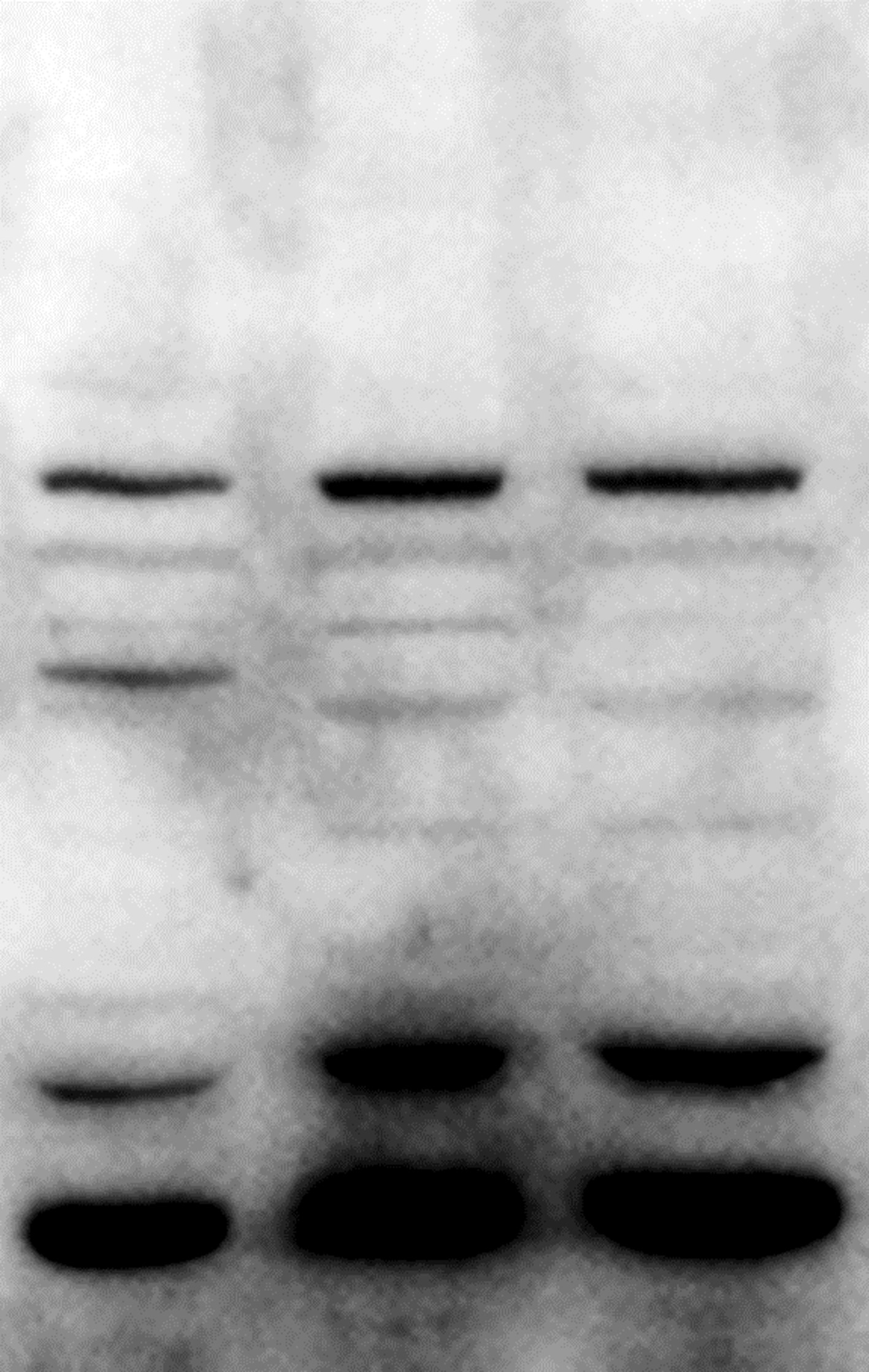

Supplement: Figure 2—figure supplement 1—source data 4. [file elife-105318-fig2-figsupp1-data4.zip › Figure 2-supplement figure 2B-source data/Figure 2-supplement figure 2b-source data 1.jpg]

Py10-LDHA →

| 6133                                                                              | 6133<br>+v1                                                                       | 6133<br>+v2                                                                         |
|-----------------------------------------------------------------------------------|-----------------------------------------------------------------------------------|-------------------------------------------------------------------------------------|
| 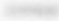 | 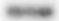 | 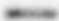 |

6133

6133  
+v1

6133  
+v2

Supplement: Figure 3—source data 1. [file elife-105318-fig3-data1.zip › Figure 3 labled source data/Figure 3-source data 7.pdf]

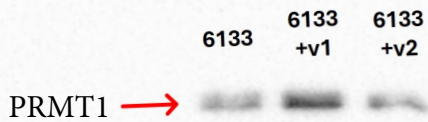

Supplement: Figure 3—source data 1. [file elife-105318-fig3-data1.zip › Figure 3 labled source data/Figure 3-source data 6.pdf]

6133      6133      6133  
         +v1      +v2

→  
LDHA

6133

6133  
+v1

6133  
+v2

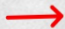

LDHA

Supplement: Figure 3—source data 1. [file elife-105318-fig3-data1.zip › Figure 3 labled source data/Figure 3-source data 5.pdf]

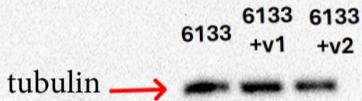

Supplement: Figure 3—source data 1. [file elife-105318-fig3-data1.zip › Figure 3 labled source data/Figure 3-source data 8.pdf]

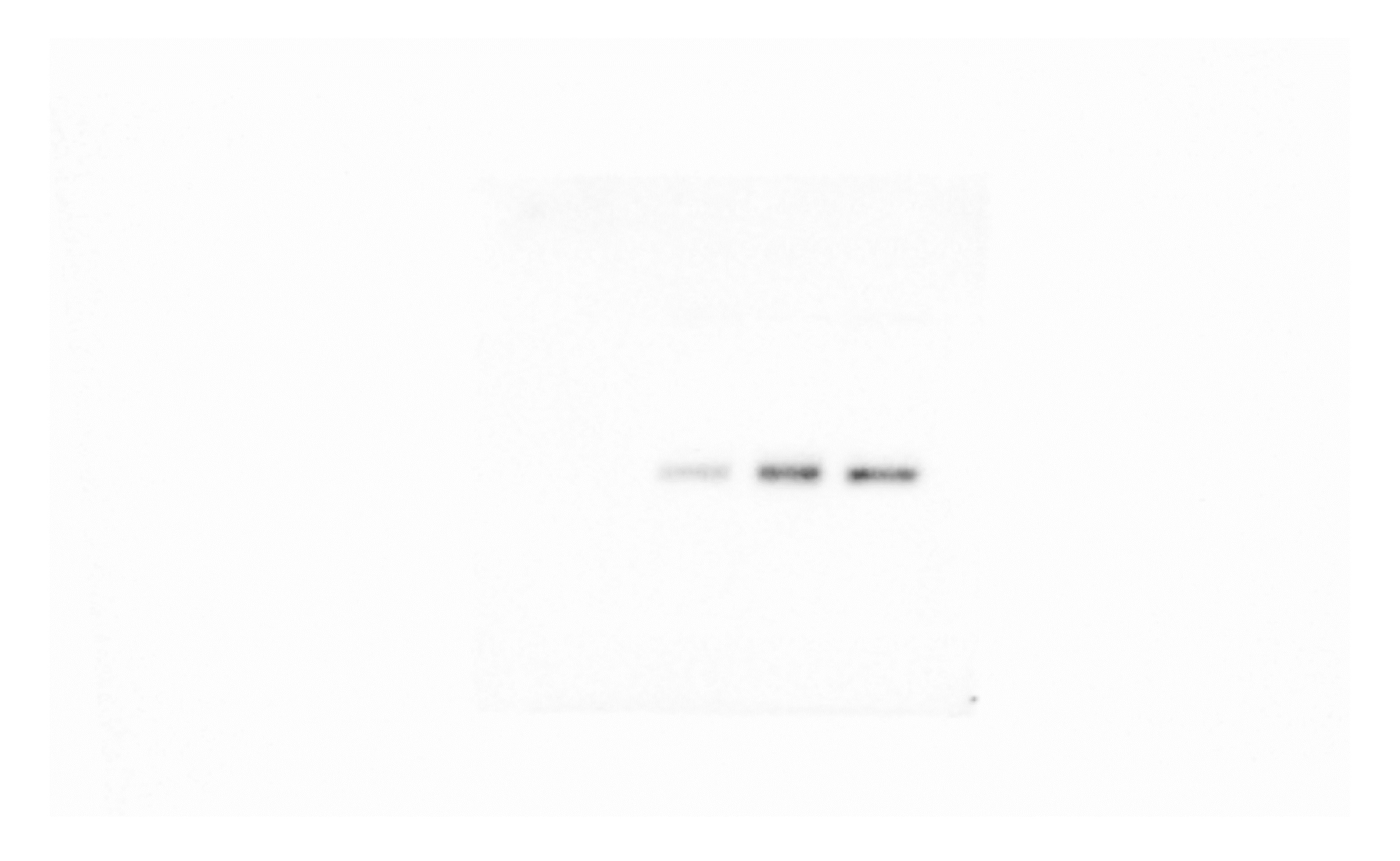

Supplement: Figure 3—source data 2. [file elife-105318-fig3-data2.zip › Figure 3 raw data/Figure 3-source data 3.jpg]

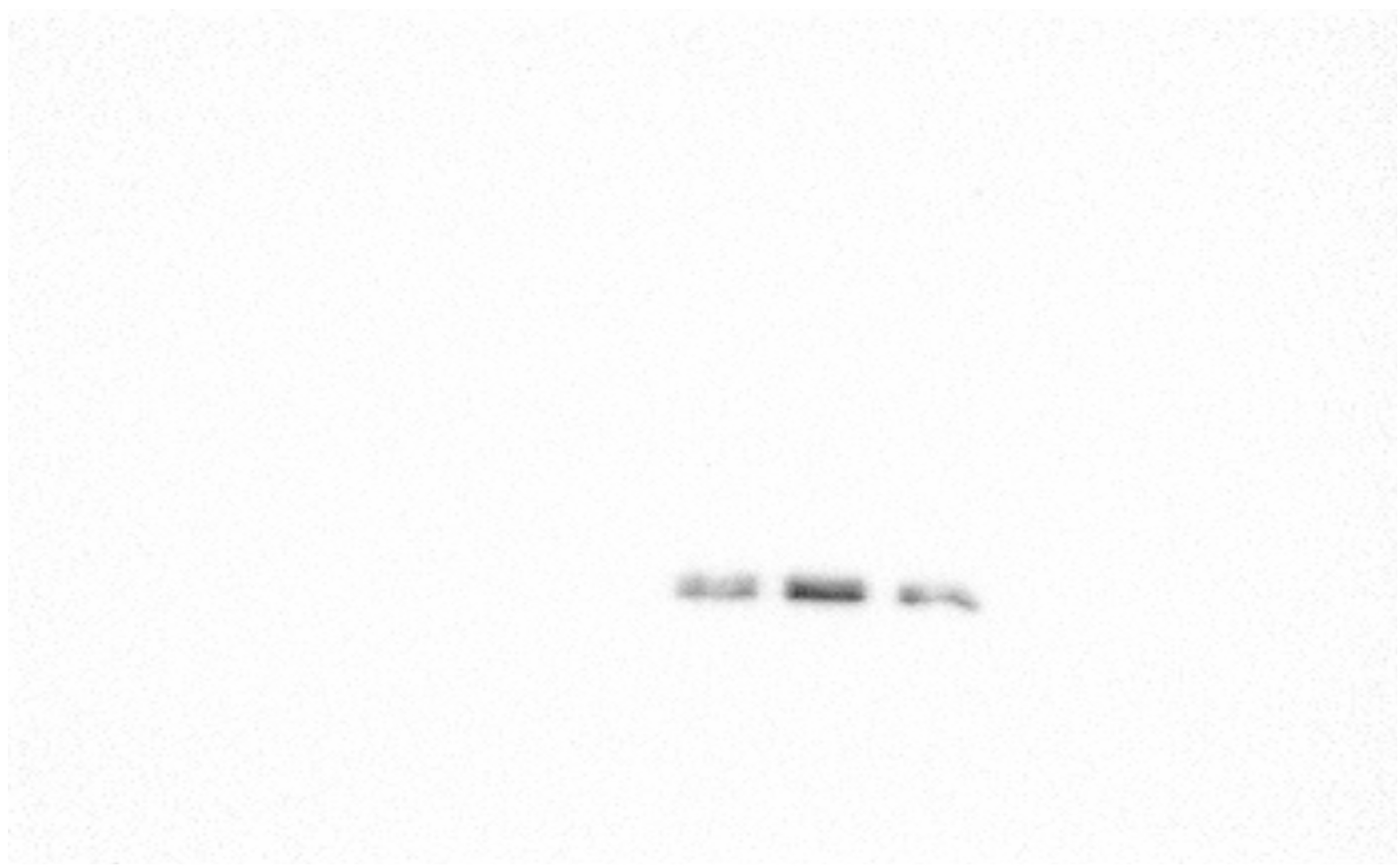

Supplement: Figure 3—source data 2. [file elife-105318-fig3-data2.zip › Figure 3 raw data/Figure 3-source data 2.jpg]

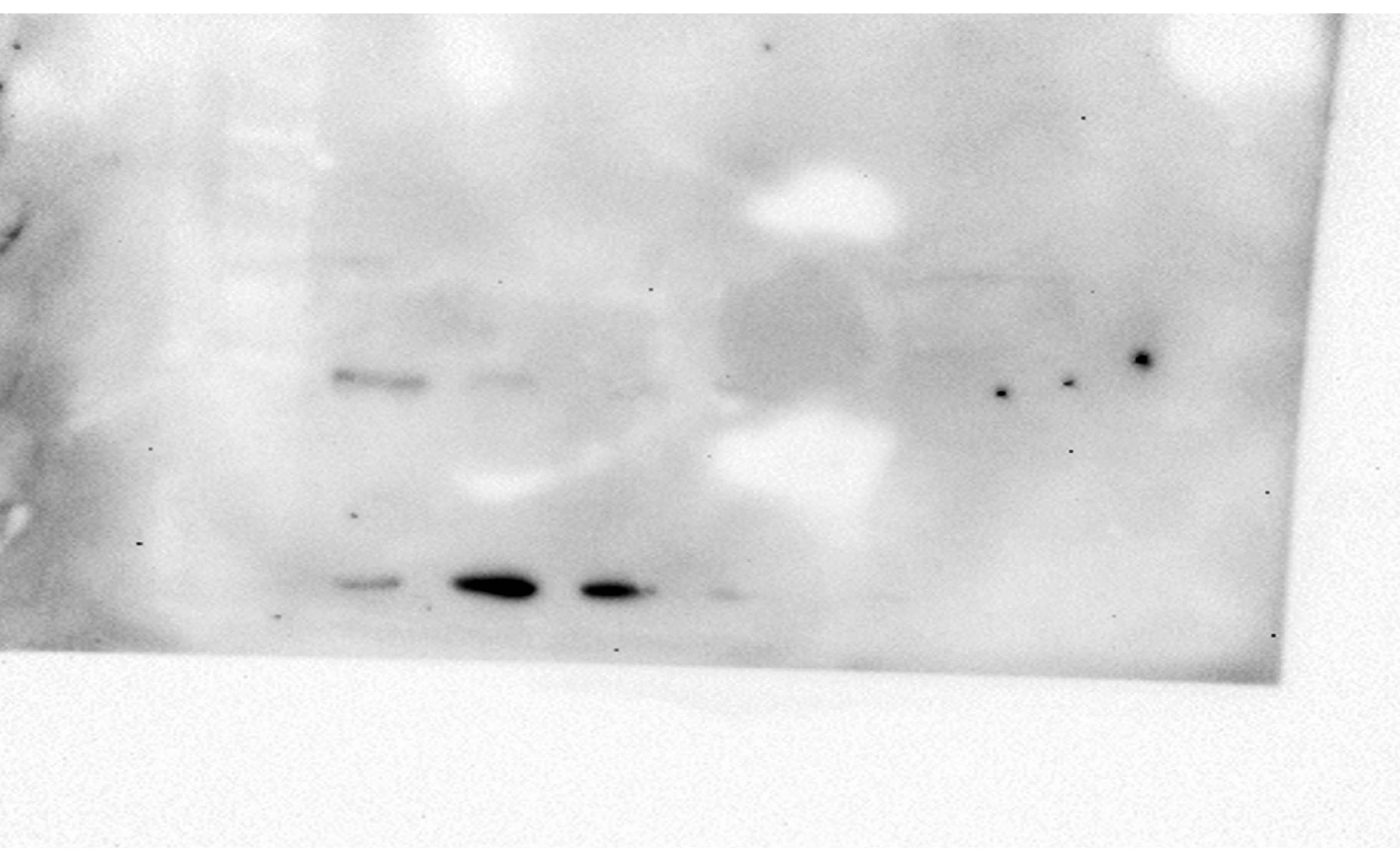

Supplement: Figure 3—source data 2. [file elife-105318-fig3-data2.zip › Figure 3 raw data/Figure 3-source data 1.jpg]

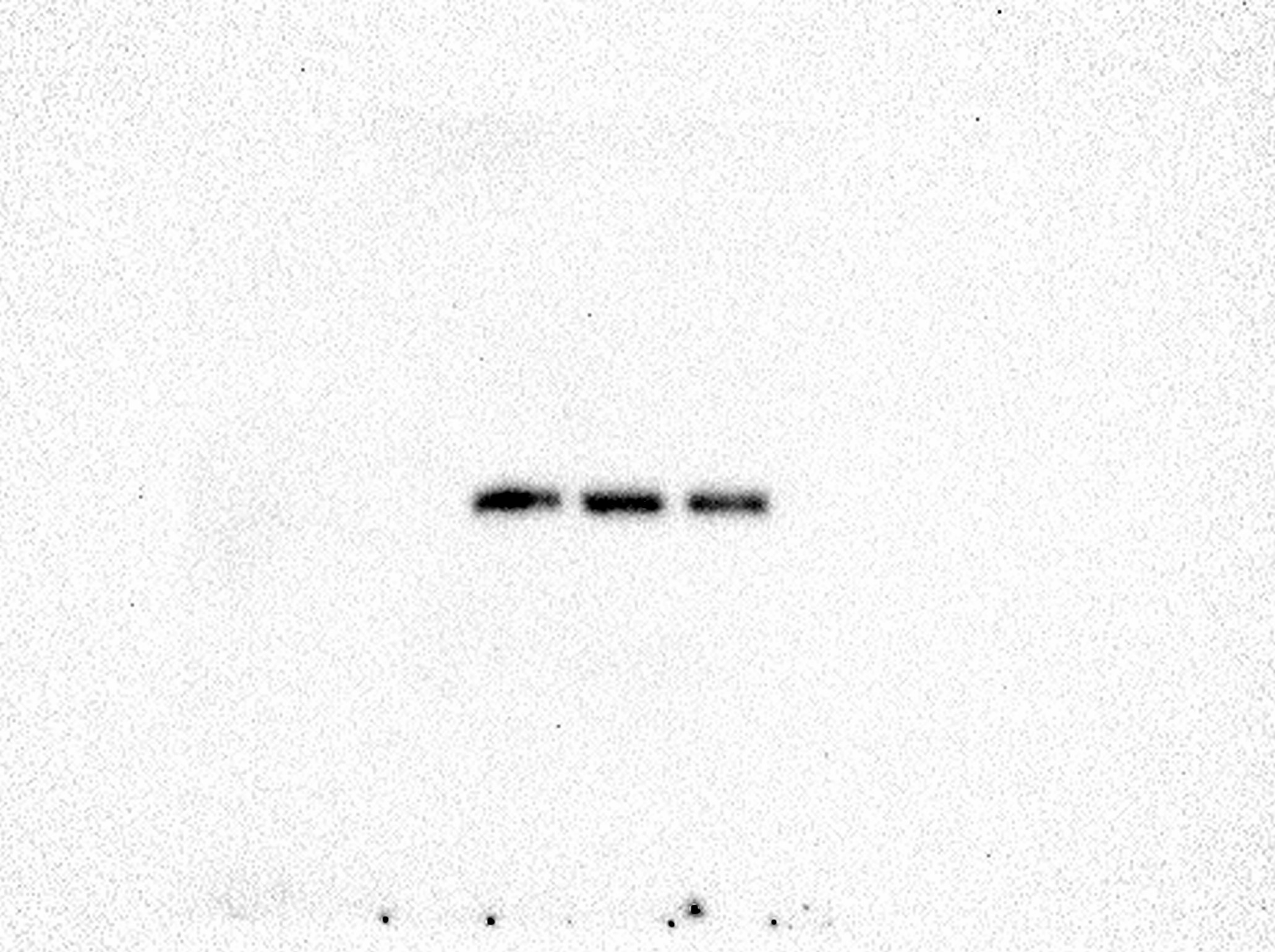

Supplement: Figure 3—source data 2. [file elife-105318-fig3-data2.zip › Figure 3 raw data/figure 3-source data 4.jpg]

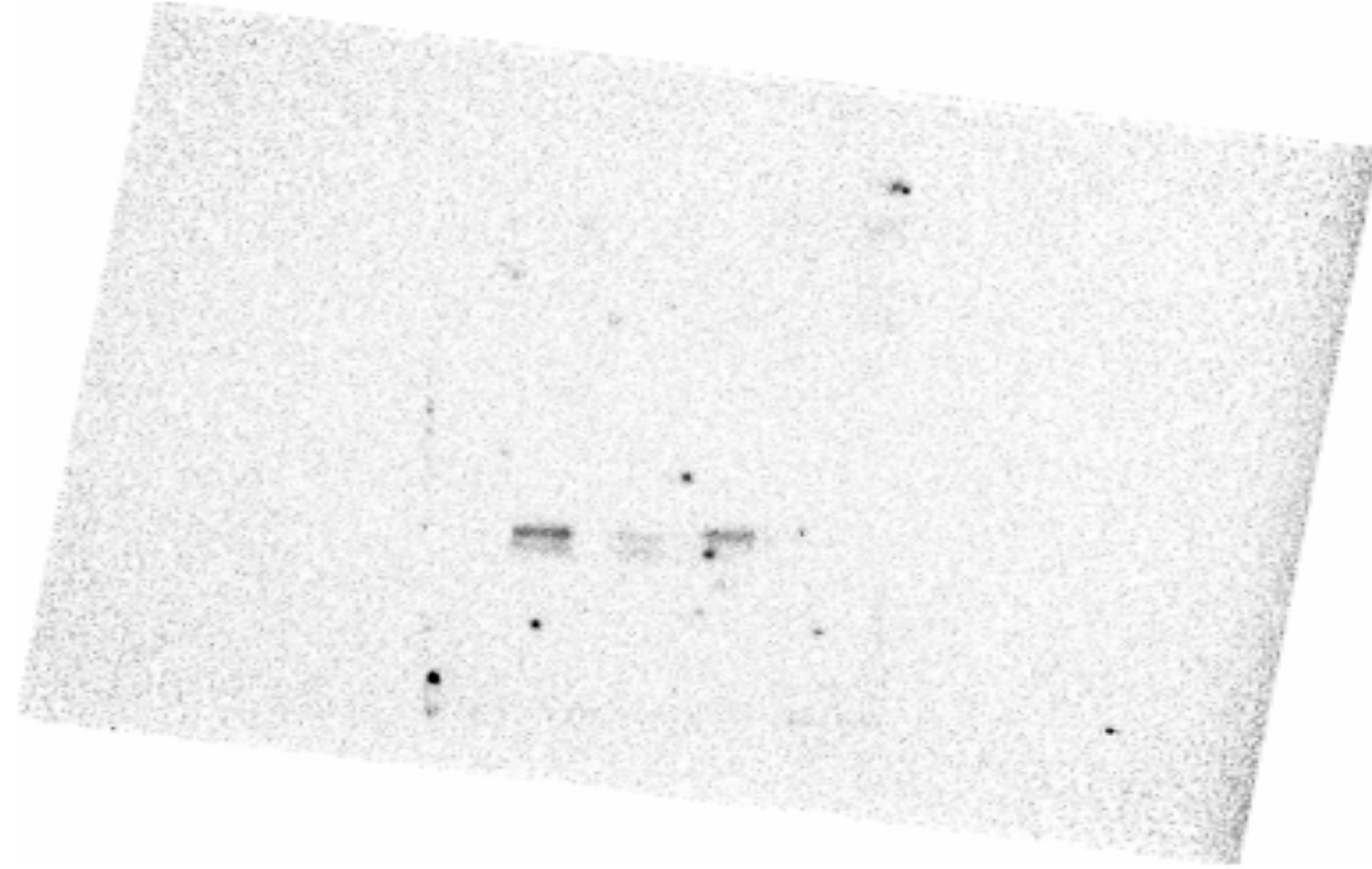

Supplement: Figure 7—source data 1. [file elife-105318-fig7-data1.zip › Figure 7 raw data/Figure 7-source data 1.jpg]

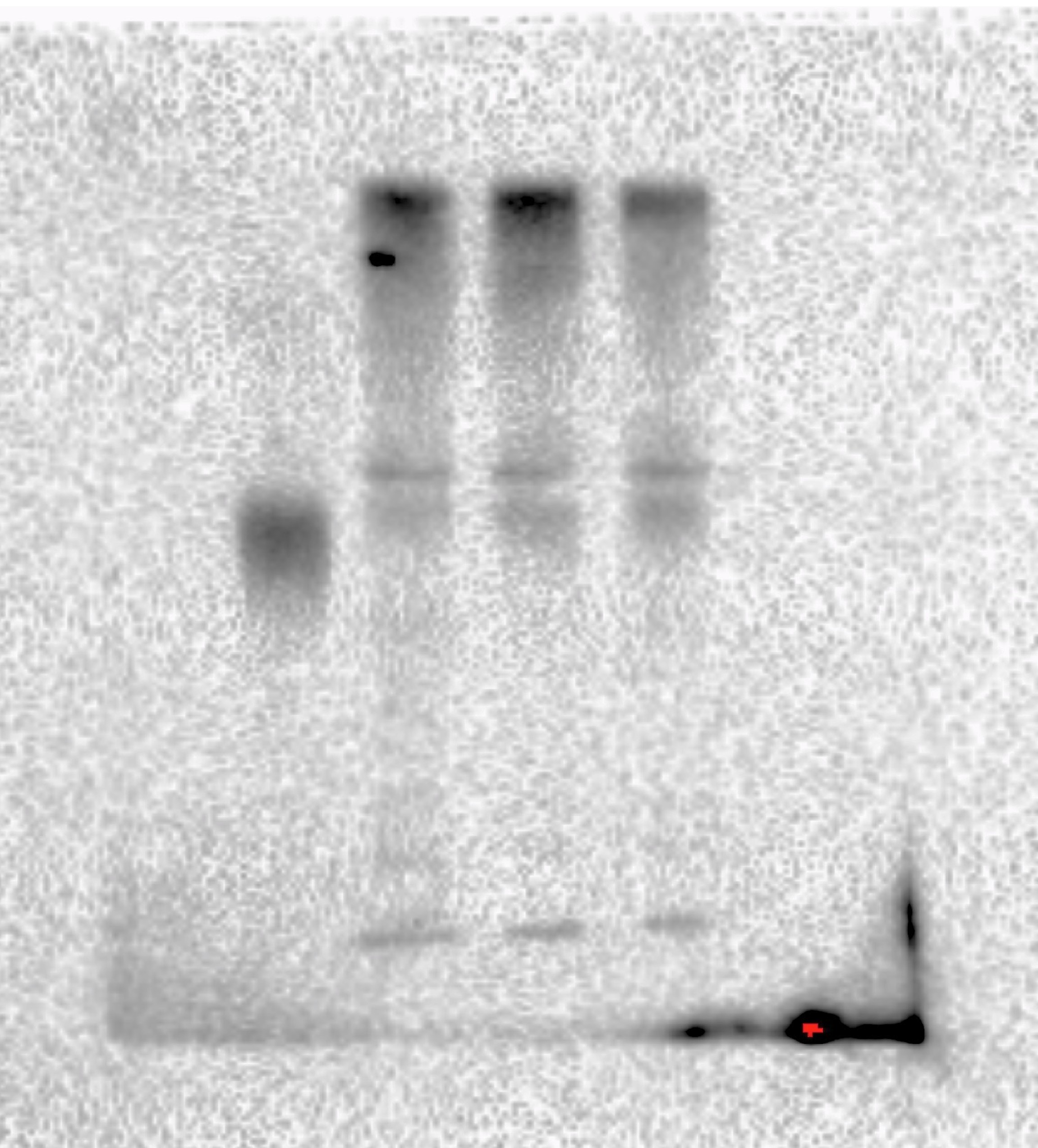

Supplement: Figure 7—source data 1. [file elife-105318-fig7-data1.zip › Figure 7 raw data/Figure 7-source data 3.jpg]

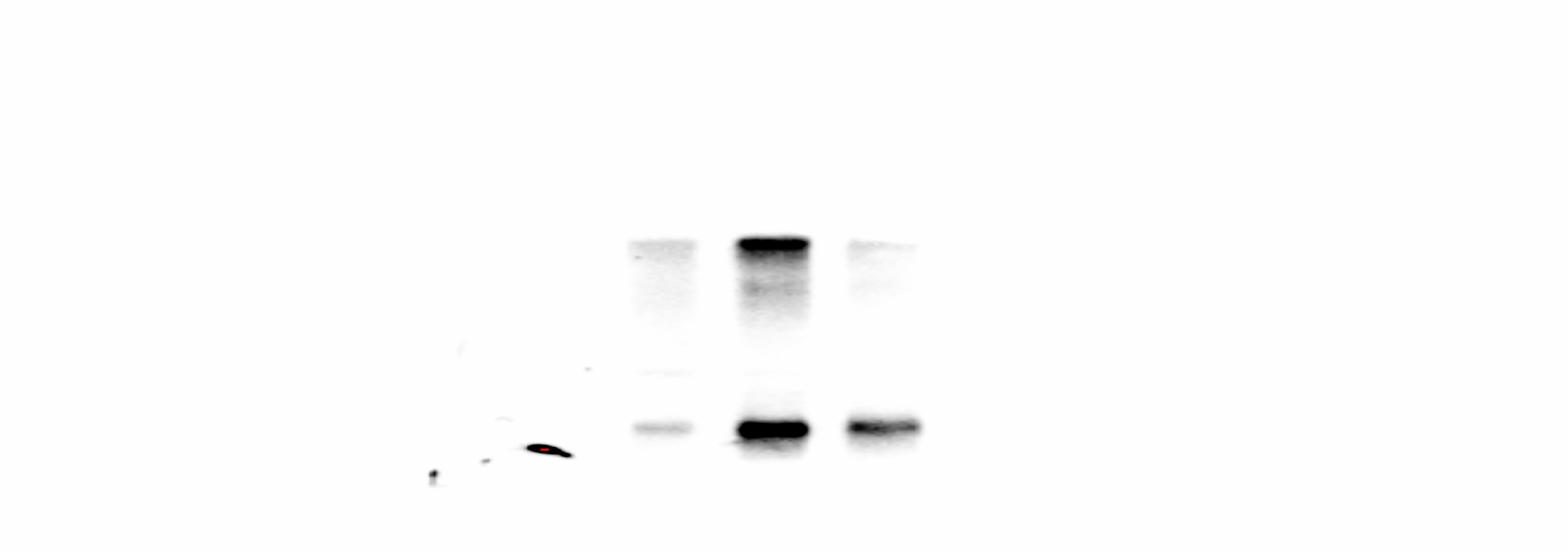

Supplement: Figure 7—source data 1. [file elife-105318-fig7-data1.zip › Figure 7 raw data/Figure 7-source data 2.jpg]
